# Prenatal Bisphenol A Exposure and Sex‐Differentiated Childhood BMI Over Time: A Longitudinal Korean Cohort Study

**DOI:** 10.1111/ijpo.70129

**Published:** 2026-07-05

**Authors:** Youn‐Hee Lim, Yun‐Chul Hong, Yun Jeong Lee, Choong Ho Shin, Dongwook Lee, Bung‐Nyun Kim, Johanna Inhyang Kim, Young Ah Lee

**Affiliations:** ^1^ Section of Environmental Health, Department of Public Health University of Copenhagen Copenhagen Denmark; ^2^ Department of Preventive Medicine Seoul National University College of Medicine Seoul Republic of Korea; ^3^ Environmental Health Center Seoul National University College of Medicine Seoul Republic of Korea; ^4^ Institute of Environmental Medicine Seoul National University Medical Research Center Seoul Republic of Korea; ^5^ Department of Pediatrics Seoul National University Children's Hospital Seoul Republic of Korea; ^6^ Division of Children and Adolescent Psychiatry, Department of Psychiatry Seoul National University Hospital Seoul Republic of Korea; ^7^ Department of Psychiatry Hanyang University College of Medicine Seoul Republic of Korea

**Keywords:** bisphenol A, body mass index, prenatal effects, sex‐specific effect

## Abstract

**Background:**

Prenatal exposure to bisphenol A (BPA), an endocrine‐disrupting chemical, may influence childhood obesity. Evidence on sex‐specific effects remains inconsistent.

**Methods:**

We analysed 528 mother–child pairs from a Korean birth cohort. Maternal urinary BPA concentrations were measured during mid‐pregnancy. Children's BMI and BMI Z‐score were assessed at ages 2, 4, 6, 8, and 10 years. Associations were estimated using linear and mixed‐effects models, stratified by sex and adjusted for maternal and child covariates.

**Results:**

The mean maternal BPA concentration was 2.3 μg/g creatinine. BPA exposure was positively associated with BMI and BMI Z‐score in boys but negatively associated in girls. At age 10, this divergence was most apparent. Mixed‐effects models showed a 0.13 kg/m^2^ (95% CI: 0.01, 0.25) increase in BMI among boys and a 0.20 kg/m^2^ (95% CI: −0.32, −0.08) decrease among girls per 1‐unit increase in log‐transformed BPA. Similar patterns were observed for BMI *Z*‐score.

**Conclusions:**

Prenatal BPA exposure exhibited sexually dimorphic associations with BMI from early childhood to age 10. These findings underscore the importance of considering sex‐specific effects in environmental health research and support policies to reduce BPA exposure during pregnancy.

## 1 Introduction

Bisphenol A (BPA) is a ubiquitous environmental chemical widely used in the production of polycarbonate plastics and epoxy resins, which are found in food containers, water bottles, and thermal paper. Human exposure is nearly universal, including among pregnant women, with evidence of placental transfer to the fetus [1, 2, 3, 4, 5, 6]. BPA is recognized as an endocrine‐disrupting chemical that can interfere with metabolic regulation, potentially contributing to obesity through mechanisms such as altered adipogenesis and lipid metabolism [7].

Epidemiological studies examining the relationship between prenatal BPA exposure and childhood adiposity have produced inconsistent results. Some have reported that higher maternal BPA levels are associated with increased body mass index (BMI) or adiposity measures in boys but decreased outcomes in girls, while others have observed the opposite or no clear sex‐specific pattern [8, 9, 10, 11]. This inconsistency may be partly due to variation in exposure timing, outcome definitions, and age at follow‐up [10, 12].

Experimental animal studies support the hypothesis of sex‐dimorphic metabolic effects of early BPA exposure, suggesting that BPA can interact with oestrogenic pathways and influence fat cell differentiation in a sex‐dependent manner [13, 14]. However, human evidence remains sparse and geographically limited, with most studies conducted in North America and Europe, and few following children longitudinally through critical stages of growth [8, 9, 10, 11, 12, 15, 16, 17].

To address these gaps, we conducted a prospective cohort study in South Korea to evaluate the sex‐specific associations between maternal BPA exposure during pregnancy and children's BMI and BMI *Z*‐score from early childhood through age 10. Our study offers novel insights from an Asian population and assesses whether early‐life BPA exposure differentially influences BMI in boys and girls during a key window for obesity development.

## 2 Materials and Methods

### 2.1 Population

This study used data from the Environment and Development of Children (EDC) cohort, a prospective birth cohort designed to investigate environmental risk factors for child development in South Korea. The EDC is a sub‐cohort of the larger Congenital Anomaly Study (CAS), which recruited 13 484 pregnant women from eight hospitals in Seoul and surrounding regions (Gyeonggi and Incheon) between 2008 and 2011. Of these, 10 752 women gave birth to infants without congenital anomalies. From 2012 to 2015, a random sample of 2085 of these mothers was contacted, and 675 mothers and 703 children were enrolled in the EDC cohort. Pregnant women's lifestyle and sociodemographic information were collected, and urine samples were analysed for BPA, phthalates, and 3‐Phenoxybenzoic acid, while blood and urine samples were collected for assessment of heavy metals [18]. Aside from more frequent alcohol consumption, participants included in the EDC cohort were similar to those excluded in terms of active smoking, caesarean delivery rate, maternal age, and birth weight [18]. Detailed descriptions of the study population can be found elsewhere [18].

After enrollment, children were followed every 2 years around their birthdates (±1 month). Because follow‐up began in 2012, some children who were already 2 years old in 2010–2011 could not be recontacted. At each visit, blood and urine samples were collected between 9:00 AM and 12:00 PM. Trained technicians at Seoul National University Hospital measured the children's anthropometrics and collected information on lifestyle factors, environmental exposures, food frequency, and the medical history of the child and parents using a structured questionnaire. As children grew older, additional age‐relevant exposure and health outcome measures were incorporated into follow‐up assessments [18].

Among 703 eligible children, we excluded those from multiple births (*n* = 55), born preterm (< 37 weeks of gestation; *n* = 32), with low birth weight (< 2.5 kg; *n* = 7) or macrosomia (> 4 kg; *n* = 23), missing maternal BPA measurements (*n* = 46), and missing covariate data (*n* = 12). The final analytic sample included 528 mother–child pairs (Figure S1). Children were followed at five time points, ages 2, 4, 6, 8, and 10 years, between 2012 and 2019. At each visit, informed consent was obtained from parents and, where appropriate, assent from children. The study protocol was approved by the Institutional Review Board of Seoul National University College of Medicine (IRB No. 1201–010‐392).

### 2.2 Exposure Assessment

Maternal BPA exposure was measured from spot urine samples collected in polypropylene containers during the second trimester of pregnancy (14–27 weeks of gestation). Morning urine samples were collected and stored at −70°C until analysis at a certified laboratory (SMARTIVE Co., Seoul, Korea). After enzymatic deconjugation with β‐glucuronidase/sulfatase at 37°C overnight, samples were subjected to solid‐phase extraction, and BPA was eluted with methanol. Urinary BPA concentrations were measured using high‐performance liquid chromatography–tandem mass spectrometry (Agilent 6420 Triple Quadrupole LC–MS/MS).

Quality control procedures included procedural blanks and internal QC samples in each batch. The calibration curve showed excellent linearity (*R* ^2^ = 0.999). Accuracy was verified using National Institute of Standards and Technology standard reference materials (3672 and 3673), with a BPA recovery rate of 103.3%, and intra‐ and inter‐day coefficients of variation were ≤ 5%. External QC was ensured through participation in the German External Quality Assessment Scheme.

BPA concentrations were adjusted for creatinine to account for dilution, yielding values in μg/g creatinine. BPA levels below the limit of detection (LOD; 0.212 μg/L) were imputed using the LOD divided by the square root of 2. BPA concentrations were natural log‐transformed to approximate a normal distribution prior to analysis.

### 2.3 Anthropometric Measurements

At each follow‐up visit, trained staff measured height using a Harpenden stadiometer and weight using a digital scale (150A; CAS Co. Ltd., Seoul, Korea). BMI was calculated as weight (kg) divided by height squared (m^2^). BMI *Z*‐score was computed using the 2007 Korean National Growth Charts [19]. Overweight status in children was defined as their BMI being at or above the 85th percentile for age and sex.

### 2.4 Covariates

Maternal covariates included age at delivery, parity (nulliparous or multiparous), smoking status during pregnancy (current, past, or never), educational attainment (< college or ≥ college), and pre‐pregnancy BMI. Child‐specific covariates included birth year, age at visit (months), sex, gestational age, birth weight, and breastfeeding for at least 6 months postpartum. These were collected via structured questionnaires and medical records at baseline.

### 2.5 Statistical Analysis

To visualize mean BMI and BMI *Z*‐score over time by maternal BPA exposure, we calculated sex‐specific means for BMI and BMI *Z*‐score within maternal BPA exposure groups: low (< geometric median [1.32 μg/g creatinine]) and high (≥ geometric median).

To guide covariate selection and control for potential confounding, we constructed a directed acyclic graph (DAG) and identified a minimally sufficient adjustment set for each model (see Figure S2).

At each follow‐up age, we assessed associations between natural log‐transformed maternal BPA concentrations and children's BMI and BMI *Z*‐score using linear regression models and children's overweight status using logistic regression models. All associations were expressed per one‐unit increase in natural log‐transformed maternal BPA concentrations. Crude models adjusted for child age and sex. Fully adjusted models included maternal age, pre‐pregnancy BMI, parity, educational attainment, smoking status, and child‐specific factors such as birth year, age at visit (months), sex, gestational age, and birth weight. Sex‐stratified analyses were conducted to examine potential differences in associations between maternal BPA exposure and BMI and BMI *Z*‐score in boys and girls.

To assess longitudinal trends, we used mixed‐effects models with a random intercept for each child to estimate average changes in BMI and BMI *Z*‐score across all follow‐up ages. To formally test for sex‐specific differences, we included interaction terms between BPA exposure and child sex.

We conducted several sensitivity analyses. Given that breastfeeding is protective against childhood obesity [20], we conducted a sensitivity analysis adjusting for breastfeeding duration (> 6 months vs. ≤ 6 months) as a covariate. We examined potential non‐linear associations between prenatal BPA exposure and BMI or BMI *Z*‐score across all follow‐up ages by modelling BPA exposure as a categorical variable divided into quartiles. Finally, we calculated the mediation proportion from a mediation analysis to assess the indirect effect of prenatal BPA exposure on a child's BMI via birth weight, stratified by age and sex.

All effect estimates were reported with corresponding 95% confidence intervals (CIs) to assess statistical precision. Analyses were performed using R version 4.4.2.

## 3 Results

### 3.1 Participant Characteristics

The final sample included 528 mother–child pairs (273 boys and 255 girls). The mean maternal age at enrollment was 31.2 years. The cohort was of relatively high socioeconomic status overall; 59.1% of mothers were nulliparous, only 15.9% had less than a college education, and 69.6% reported a monthly household income ≥ 4 000 000 KRW. In addition, approximately 54.2% were never‐smokers, and 48.1% exclusively breastfed for at least 6 months postpartum (Table 1).

**TABLE 1:** Characteristics of the mothers in the study [mean ± standard deviation (SD), *n* (%)].

Variables	Overall	Boys	Girls	*p*‐value*
Number of mothers	528	273	255	
Maternal age (years)	31.2 ± 3.6	31.3 ± 3.7	31.0 ± 3.5	0.29
Gestational age (days)	275.8 ± 7.4	275.2 ± 7.6	276.5 ± 7.1	0.05
Birth weight (g)	3278.0 ± 339.7	3323.7 ± 323.6	3228.9 ± 350.3	< 0.01
Pre‐pregnancy BMI (kg/m^2^)	22.0 ± 2.9	21.9 ± 2.9	22.0 ± 2.9	0.88
Education < college	84 (15.9)	43 (15.8)	41 (16.1)	1.00
Never smoker	286 (54.2)	140 (51.3)	146 (57.3)	0.20
Nulliparous	312 (59.1)	162 (59.3)	150 (58.8)	0.97
Income ≥ 4 000 000 KRW	297 (69.6)	148 (64.9)	149 (74.9)	0.03
Breast feeding > 6 months	254 (48.1)	130 (47.6)	124 (48.6)	0.89

Abbreviation: BMI, body mass index.

* *p*‐values were derived from chi‐square tests and *t*‐tests for categorical and continuous variables by sex.

Children's BMI and BMI *Z*‐score increased with age: mean BMI rose from 16.5 (SD: 1.4) at age 2 to 18.7 (SD: 3.2) at age 10, while BMI Z‐score increased from −0.2 (SD: 0.9) to 0.2 (SD: 1.0) over the same period. The prevalence of overweight increased from 6.6% at age 2 to 25.0% at age 10 (Table 2:). BPA was detected in 89.4% of maternal samples. Mid‐pregnancy maternal BPA concentrations had an arithmetic mean of 2.3 μg/g creatinine (geometric mean: 1.4 μg/g creatinine), and were higher among mothers of boys than among mothers of girls (2.6 vs. 2.0 μg/g creatinine, *p* = 0.02) (Table 3). From age 2 to 10 years, mean BMI and BMI Z‐score were consistently higher among boys with higher maternal BPA exposure (i.e., ≥ geometric median), whereas girls showed the opposite pattern, with higher exposure consistently associated with lower BMI and BMI *Z*‐score (Figure S3).

**TABLE 2:** Characteristics of the children in the study [mean ± standard deviation (SD), *n* (%)].

		Age 2	Age 4	Age 6	Age 8	Age 10
Total	Number of children	304	478	427	391	340
	Age (months)	23.3 ± 0.8	47.2 ± 1.6	71.2 ± 1.6	95.1 ± 1.3	119.2 ± 1.8
	Weight (kg)	12.3 ± 1.3	16.2 ± 1.9	21.2 ± 3.3	27.6 ± 5.2	36.9 ± 7.8
	Height (cm)	86.2 ± 3.0	101.9 ± 3.8	115.6 ± 4.4	127.9 ± 4.9	139.9 ± 5.7
	BMI (kg/m^2^)	16.5 ± 1.4	15.6 ± 1.3	15.8 ± 1.8	16.8 ± 2.5	18.7 ± 3.2
	BMI Z‐score	−0.2 ± 0.9	−0.1 ± 1.1	−0.1 ± 1.1	0.0 ± 1.1	0.2 ± 1.0
	Overweight	20 (6.6)	59 (12.3)	58 (13.6)	65 (16.6)	85 (25.0)
Boys	Number of children	154	253	228	181	185
	Age (months)	23.3 ± 0.8	47.2 ± 1.7	71.1 ± 1.6	95.0 ± 1.3	119.1 ± 1.6
	Weight (kg)	12.6 ± 1.3	16.4 ± 1.8	21.4 ± 3.2	28.1 ± 5.3	38.1 ± 8.1
	Height (cm)	86.8 ± 2.9	102.4 ± 3.8	116.2 ± 4.5	128.5 ± 4.7	140.0 ± 5.4
	BMI (kg/m^2^)	16.8 ± 1.4	15.7 ± 1.2	15.8 ± 1.7	16.9 ± 2.5	19.3 ± 3.3
	BMI *Z*‐score	−0.1 ± 0.9	−0.2 ± 1.0	−0.2 ± 1.0	0.0 ± 1.0	0.3 ± 1.0
	Overweight	13 (8.4)	27 (10.7)	23 (10.1)	29 (13.8)	51 (27.6)
Girls	Number of children	150	225	199	210	155
	Age (months)	23.3 ± 0.7	47.3 ± 1.6	71.2 ± 1.5	95.2 ± 1.4	119.4 ± 1.9
	Weight (kg)	11.9 ± 1.2	16.0 ± 2.0	21.0 ± 3.4	26.9 ± 5.0	35.5 ± 7.2
	Height (cm)	85.6 ± 2.9	101.3 ± 3.7	115.1 ± 4.2	127.2 ± 5.0	139.8 ± 6.0
	BMI (kg/m^2^)	16.2 ± 1.3	15.6 ± 1.4	15.8 ± 2.0	16.6 ± 2.4	18.0 ± 2.9
	BMI Z‐score	−0.2 ± 0.9	−0.1 ± 1.1	0.0 ± 1.1	0.0 ± 1.1	0.1 ± 1.1
	Overweight	7 (4.7)	32 (14.2)	35 (17.6)	36 (19.9)	34 (21.9)

Abbreviations: BMI, body mass index; BMI‐Z, BMI Z‐score.

**TABLE 3:** Maternal urinary concentration levels of bisphenol A (BPA) in the study (*n* = 528).

	Detection frequency above LOD (0.212 μg/L), %	Maternal BPA concentration level, μg/g creatinine			
		Arithmetic mean ± SD	*p*‐value^a^	Geometric mean ± SD	*p*‐value^a^
Overall	89.4%	2.3 ± 3.2		1.4 ± 2.8	
Boys' mothers	90.8%	2.6 ± 3.8	0.02	1.5 ± 2.9	0.03
Girls' mothers	87.8%	2.0 ± 2.3		1.2 ± 2.7	

^a^ *p*‐values were derived from *t*‐tests assessing differences in maternal BPA concentration levels by sex; SD: standard deviation.

### 3.2 Main Associations

**FIGURE 1:**
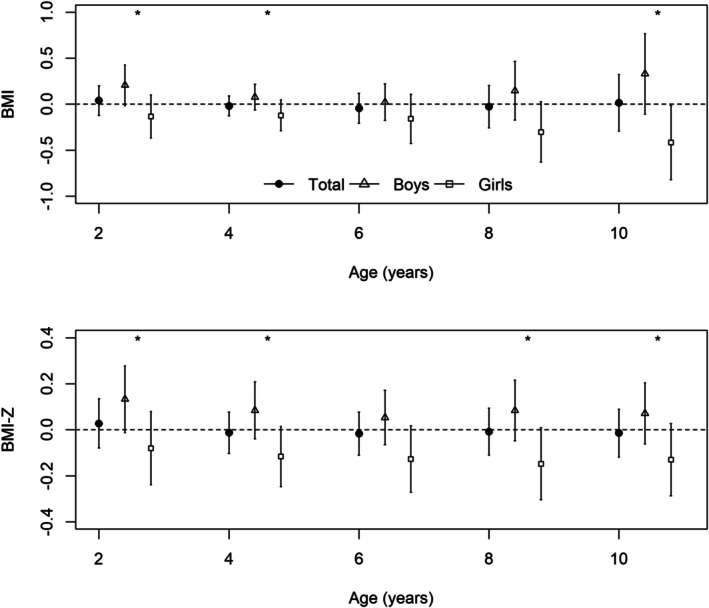
Sex‐stratified changes in BMI and BMI Z‐score (95% confidence intervals) at each age associated with a one‐unit increase in the log‐transformed maternal BPA exposure (*n* = 528). BMI, body mass index; BMI‐Z, BMI *Z*‐score; BPA, bisphenol A. *p‐value < 0.05 for interaction by sex.

In the overall sample, we observed no significant association between maternal BPA exposure and BMI or BMI Z‐score at each age when not stratified by sex (solid circles in Figure 1). However, sex‐stratified analyses revealed contrasting patterns.

Although most associations were not statistically significant, higher maternal BPA levels were associated with increases in both BMI and BMI Z‐score among boys, whereas among girls, BPA exposure was associated with decreases in BMI and BMI Z‐score. For example, at age 2, a 1‐unit increase in log‐transformed maternal BPA concentration was associated with a 0.13‐unit increase in BMI Z‐score for boys (95% CI: −0.01, 0.28) and a −0.08‐unit change for girls (95% CI: −0.24, 0.08), with a significant sex interaction (*p* = 0.03). While similar sex‐specific differences persisted from ages 4 to 10, the positive associations in boys and negative associations in girls became more pronounced with age (Figure 1, Table S1). In a sensitivity analysis examining quartiles of BPA exposure, the associations exhibited non‐monotonic patterns but showed a similar trend with more pronounced changes among girls: higher maternal BPA exposure was associated with higher BMI or BMI *Z*‐score in boys, whereas the opposite trend was observed in girls (Figure S4). Logistic regression models for overweight status showed similar sex‐differentiated trends, although associations were generally weaker and did not reach statistical significance (Table S2).

In mixed‐effects models assessing repeated BMI measurements over time, we found significant sex‐specific associations. Among boys, a 1‐unit increase in log‐transformed maternal BPA was associated with a 0.15 kg/m^2^ increase in BMI (95% CI: 0.03, 0.27), while among girls, the association was −0.20 kg/m^2^ (95% CI: −0.33, −0.07). For BMI *Z*‐score, the corresponding estimates were 0.08 (95% CI: 0.02, 0.14) in boys and −0.12 (95% CI: −0.18, −0.05) in girls (Table 4). The results remained unchanged after additional adjustment for breastfeeding (Table S3).

**TABLE 4:** Sex‐stratified changes in longitudinal BMI and BMI *Z*‐scores (95% confidence intervals) associated with a one‐unit increase in the log‐transformed maternal BPA exposure (*n* = 528).

Sex	BMI (kg/m^2^)	BMI *Z*‐score
Overall^a^	−0.01 (−0.10, 0.08)	−0.01 (−0.05, 0.04)
Boys^a^	0.15 (0.03, 0.27)*	0.08 (0.02, 0.14)*
Girls^a^	−0.20 (−0.33, −0.07)*	−0.12 (−0.18, −0.05)*

*Note:* Models were adjusted for maternal characteristics, including age, parity, pre‐pregnancy BMI, educational attainment, and smoking status, as well as child characteristics such as birth year, age at visit (months), sex (available only in overall estimates), gestational age, and birth weight.

Abbreviations: BMI, body mass index; BMI‐Z, BMI Z‐score; BPA, bisphenol A.

^a^ Associations for overall ages were analysed using linear mixed models.

* *p*‐value < 0.05.

In the mediation analyses stratified by age and sex, we found no statistically significant mediation effects of birth weight on the association between maternal BPA exposure and child BMI. Nevertheless, birth weight accounted for 24.3% (*p* = 0.24) and 35.8% (*p* = 0.81) of the association with BMI among boys at ages 4 and 6, respectively, and for 25.2% (*p* = 0.27) of the association among girls at age 2. We did not observe any meaningful mediation of BMI at other ages by birth weight in relation to maternal BPA exposure (Table S4).

## 4 Discussion

In this prospective cohort study of Korean children, we observed sex‐specific associations between maternal BPA exposure during pregnancy and childhood BMI over time. Specifically, higher maternal BPA concentrations were linked to increased BMI and BMI *Z*‐score in boys, while girls showed inverse associations. These divergent patterns were consistent across multiple age points.

Our findings align with prior research suggesting that BPA may exert sex‐dimorphic effects on metabolic outcomes. Several studies have reported similar patterns, where maternal BPA exposure was positively associated with adiposity in boys but negatively associated or null in girls during early to middle childhood [8, 9, 11]. For example, Vafeiadi et al. found that prenatal BPA levels were positively associated with adiposity measures in boys, but negatively in girls in Greece [8], and two US studies similarly reported that negative associations appeared stronger in girls than in boys [9, 11]. However, conflicting results also exist: Braun et al. reported the opposite pattern, with BPA‐associated adiposity increases observed in girls, but not boys [10], while Hoepner et al. found no evidence of sex‐specific effects [12]. These discrepancies may stem from differences in study design, BPA measurement timing, exposure levels, population characteristics, or outcome definitions.

The biological plausibility of our findings is supported by mechanistic and animal studies. BPA has been shown to promote adipogenesis through activation of oestrogen receptors and upregulation of key adipogenic genes. Additionally, BPA may alter the activity of 11β‐hydroxysteroid dehydrogenase type 1, enhancing lipid synthesis, particularly in male offspring [21]. In rodent models, perinatal BPA exposure has produced sex‐specific outcomes in fat accumulation, body weight, and metabolic gene expression [22]. These sex differences may be driven by hormonal regulation, epigenetic reprogramming, or differential sensitivity to endocrine disruption during development [23, 24, 25, 26].

This study contributes important longitudinal evidence from an underrepresented region. Most prior BPA studies have been conducted in North America or Europe [4, 5, 6, 8, 9, 10, 11, 12]. Maternal urinary BPA concentrations in our cohort (geometric mean: 1.4 μg/g creatinine) were comparable to those reported in pregnancy cohorts from North America and Europe. For example, a European HBM4EU compilation conducted between 2007 and 2014 reported study‐specific geometric mean BPA concentrations ranging from approximately 0.98–2.3 μg/g creatinine [27]. The HOME cohort recruited pregnant women in 2003–2006 in Ohio, US, and reported a geometric mean maternal urinary BPA concentration of 2.1 μg/g creatinine at approximately 16 weeks of gestation [28]. Differences across studies likely reflect calendar time (overall declining trends), population characteristics, and analytical approaches (e.g., creatinine/specific gravity adjustment and limits of detection). By examining Korean children ages 2–10, our study provides culturally and environmentally relevant longitudinal data and underscores the need for regional diversity in environmental health research. This long‐term follow‐up offers a rare opportunity to evaluate how prenatal BPA exposure relates to BMI across early and middle childhood.

Despite its strengths, including a well‐characterized cohort, repeated BMI measures, and rigorous adjustment for confounders, this study has limitations. First, BPA exposure was measured using a single spot urine sample during the second trimester. Given BPA's short half‐life and high intra‐individual variability [29], this may not accurately reflect long‐term exposure. This nondifferential measurement error introduced by a single BPA sample is likely to bias results toward the null. Therefore, future studies should incorporate multiple time‐point measurements or pooled samples [30].

Prenatal exposure to bisphenols and other endocrine‐disrupting chemicals commonly occurs as complex mixtures. Although data on other EDCs (e.g., phthalates) were available in this cohort, the present analysis focused on BPA to address a specific a priori hypothesis. Moreover, other bisphenols, such as Bisphenols S and F, were not measured during pregnancy. Therefore, the present study did not assess the potential combined or interactive effects of multiple EDC exposures. Future studies applying mixture‐based analytical approaches and broader exposure panels are warranted to reflect real‐world exposure scenarios. In addition, for a study with low BPA detection frequencies, it may be necessary to impute BPA concentrations using a log‐normal distribution with maximum likelihood estimation, which can provide greater variability and less biased estimates of BPA levels [31].

Second, we did not assess mechanistic biomarkers such as hormone levels, gene expression, or epigenetic changes, which limits causal inference regarding the observed sex‐specific effects. Given the limited evidence from rodent models of decreased BMI in females associated with prenatal BPA exposure, the lack of sex‐stratified associations, and the absence of statistically significant sex‐stratified mediation effects, the inverse associations observed in girls should be interpreted cautiously. These patterns may reflect sex‐specific growth trajectories, non‐monotonic endocrine‐disrupting effects, or residual confounding rather than a true protective effect of BPA.

Third, while our models adjusted for many covariates, residual confounding from unmeasured factors (e.g., maternal consumption of soda, fast food, and highly processed or packaged foods, or household exposures) cannot be excluded.

Finally, our study population was drawn from an urban cohort in Seoul and surrounding metropolitan areas (Gyeonggi and Incheon) and was characterized by relatively high socioeconomic status, including higher maternal education and household income. This may limit the generalizability of our findings to other regions of South Korea, particularly rural or socioeconomically disadvantaged populations. Differences in BPA exposure sources and health‐related factors may yield distinct exposure‐outcome relationships, and our findings should therefore be interpreted within this urban context.

## 5 Conclusion

Our findings provide evidence of sex‐specific associations between prenatal BPA exposure and BMI development in children, with opposing directions observed in boys and girls. These results highlight the importance of considering sex as a biological variable in environmental exposure research and may inform regulatory efforts aimed at reducing BPA exposure during pregnancy. Further longitudinal and mechanistic studies are needed to understand the developmental pathways linking early BPA exposure to obesity risk in offspring.

*p‐value for the interaction between maternal BPA and sex on BMI and BMI Z‐score. Models were adjusted for maternal characteristics, including age, parity, pre‐pregnancy BMI, educational attainment, and smoking status, as well as child characteristics such as birth year, age at visit (months), sex (available only in overall estimates), gestational age, and birth weight.

## Author Contributions

All authors contributed to the study conception and design. Material preparation, data collection and analysis were performed by Youn‐Hee Lim, Young Ah Lee, and Yun‐Chul Hong. The first draft of the manuscript was written by Youn‐Hee Lim and all authors commented on previous versions of the manuscript. All authors read and approved the final manuscript.

## Funding

This study was partially supported by grants from the Environmental Health Center funded by the Korean Ministry of Environment, an R&D Research program funded by the Ministry of Food and Drug Safety (#15162MFDS046), and the Basic Science Research Program through the National Research Foundation of Korea funded by the Ministry of Education (2018R1D1A1B07043446 and 2018R1D1A1B07049806).

## Ethics Statement

The study protocol was approved by the Institutional Review Board, College of Medicine, Seoul National University (IRB No. 1201–010‐392).

## Consent

Written informed consent was obtained from participants and their parents at each visit in line with the study protocol.

## Conflicts of Interest

The authors declare no conflicts of interest.

## Supporting information


**Figure S1:** Flow diagram describing the inclusion criteria of subjects enrolled in the study.
**Figure S2:** Directed acyclic graph to determine covariates in the models (maternal exposure to bisphenol A [BPA] and body mass index [BMI]).
**Figure S3:** Sex‐specific mean (a) BMI and (b) BMI‐*Z* at each age by maternal BPA exposure groups (low [below median] and high [above median]).
**Figure S4:** Sex‐specific differences in BMI and BMI *Z*‐score at each age associated with quartile‐based maternal BPA exposure (95% confidence intervals) among children (*n* = 528), with Q2 Q4 compared to Q1.
**Table S1:** Sex‐stratified changes in BMI and BMI Z‐score (95% Confidence Intervals) at each age associated with a one‐unit increase in the log‐transformed maternal BPA exposure (*n* = 528), as shown in Figure 1.
**Table S2:** Sex‐stratified odds ratio of being overweight (95% Confidence Intervals) at each age associated with a one‐unit increase in the log‐transformed maternal BPA exposure (*n* = 528).
**Table S3:** Sex‐stratified changes in longitudinal BMI and BMI *Z*‐score (95% Confidence Intervals) associated with a one‐unit increase in the log‐transformed maternal BPA exposure, before and after adjusting for breastfeeding (*n* = 528).

## Data Availability

The data that support the findings of this study are available on request from the corresponding author. The data are not publicly available due to privacy or ethical restrictions.
