# Supplementary material for: Prenatal Bisphenol A Exposure and Sex‐Differentiated Childhood BMI Over Time: A Longitudinal Korean Cohort Study
Source: Pediatr Obes. 2026 Jul 5;21(7):e70129. doi: 10.1111/ijpo.70129 (PMC13333248; doi:10.1111/ijpo.70129)
Supplement: Supplementary file 1 — Figure S1: Flow diagram describing the inclusion criteria of subjects enrolled in the study. Figure S2: Directed acyclic graph to determine covariates in the models (maternal exposure to bisphenol A [BPA] and body mass index [BMI]). Figure S3: Sex‐specific mean (a) BMI and (b) BMI‐Z at each age by maternal BPA exposure groups (low [below median] and high [above median]). Figure S4: Sex‐specific differences in BMI and BMI Z‐score at each age associated with quartile‐based maternal BPA exposure (95% confidence intervals) among children (n = 528), with Q2 Q4 compared to Q1. Table S1: Sex‐stratified changes in BMI and BMI Z‐score (95% Confidence Intervals) at each age associated with a one‐unit increase in the log‐transformed maternal BPA exposure (n = 528), as shown in Figure 1. Table S2: Sex‐stratified odds ratio of being overweight (95% Confidence Intervals) at each age associated with a one‐unit increase in the log‐transformed maternal BPA exposure (n = 528). Table S3: Sex‐stratified changes in longitudinal BMI and BMI Z‐score (95% Confidence Intervals) associated with a one‐unit increase in the log‐transformed maternal BPA exposure, before and after adjusting for breastfeeding (n = 528). [file IJPO-21-e70129-s001.pdf]

Supplementary Materials

Prenatal Bisphenol A Exposure and Sex-Differentiated Childhood BMI over time: A Longitudinal Korean Cohort Study

Youn-Hee Lim, PhD[1,2*], Yun-Chul Hong, PhD, MD[2,3,4], Yun Jeong Lee[5], Choong Ho Shin, PhD, MD[5], Dongwook Lee, MD[2], Bung-Nyun Kim, PhD, MD[6], Johanna Inhyang Kim, PhD, MD[7], Young Ah Lee, PhD, MD[5]

[1]Section of Environmental Health, Department of Public Health, University of Copenhagen, Copenhagen, Denmark

[2]Department of Preventive Medicine, Seoul National University College of Medicine, Seoul, Republic of Korea

[3]Environmental Health Center, Seoul National University College of Medicine, Seoul, Republic of Korea

[4]Institute of Environmental Medicine, Seoul National University Medical Research Center, Seoul, Republic of Korea

[5]Department of Pediatrics, Seoul National University Children's Hospital, Seoul, Republic of Korea

[6]Division of Children and Adolescent Psychiatry, Department of Psychiatry, Seoul National University Hospital, Seoul, Republic of Korea

[7]Department of Psychiatry, Hanyang University College of Medicine, Seoul, Republic of Korea

[*]Corresponding author**:** Youn-Hee Lim, PhD, Section of Environmental Health, Department of Public Health, University of Copenhagen, Copenhagen, Denmark, Telephone: +45-24611777; E-mail: younhee.lim@sund.ku.dk

Table of Contents

Supplementary Figures

Supplementary Material, Fig S1. Flow diagram describing the inclusion criteria of subjects enrolled in the study.. 3

Supplementary Material, Fig S2. Directed acyclic graph to determine covariates in the models (maternal exposure to bisphenol A [BPA] and body mass index [BMI]) 4

Supplementary Material, Fig S3. Sex-specific mean (a) BMI and (b) BMI-Z at each age by maternal BPA exposure groups (low [below median] and high [above median]) 5

Supplementary Material, Fig S4. Sex-specific differences in BMI and BMI Z-score at each age associated with quartile-based maternal BPA exposure (95% confidence intervals) among children (n = 528), with Q2–Q4 compared to Q1. 6

Supplementary Tables

Supplementary Material, Table S1. Sex-stratified changes in BMI and BMI Z-score (95% Confidence Intervals) at each age associated with a one-unit increase in the log-transformed maternal BPA exposure (n=528), as shown in Figure 1 7

Supplementary Material, Table S2. Sex-stratified odds ratio of being overweight (95% Confidence Intervals) at each age associated with a one-unit increase in the log-transformed maternal BPA exposure (n=528).. 8

Supplementary Material, Table S3. Sex-stratified changes in longitudinal BMI and BMI Z-score (95% Confidence Intervals) associated with a one-unit increase in the log-transformed maternal BPA exposure, before and after adjusting for breastfeeding (n=528) 9

Supplementary Material, Table S4. Sex-stratified mediation proportion by birth weight on the association between maternal BPA exposure and BMI at each age... 10

Supplementary Material, Fig S1. Flow diagram describing the inclusion criteria of subjects enrolled in the study.

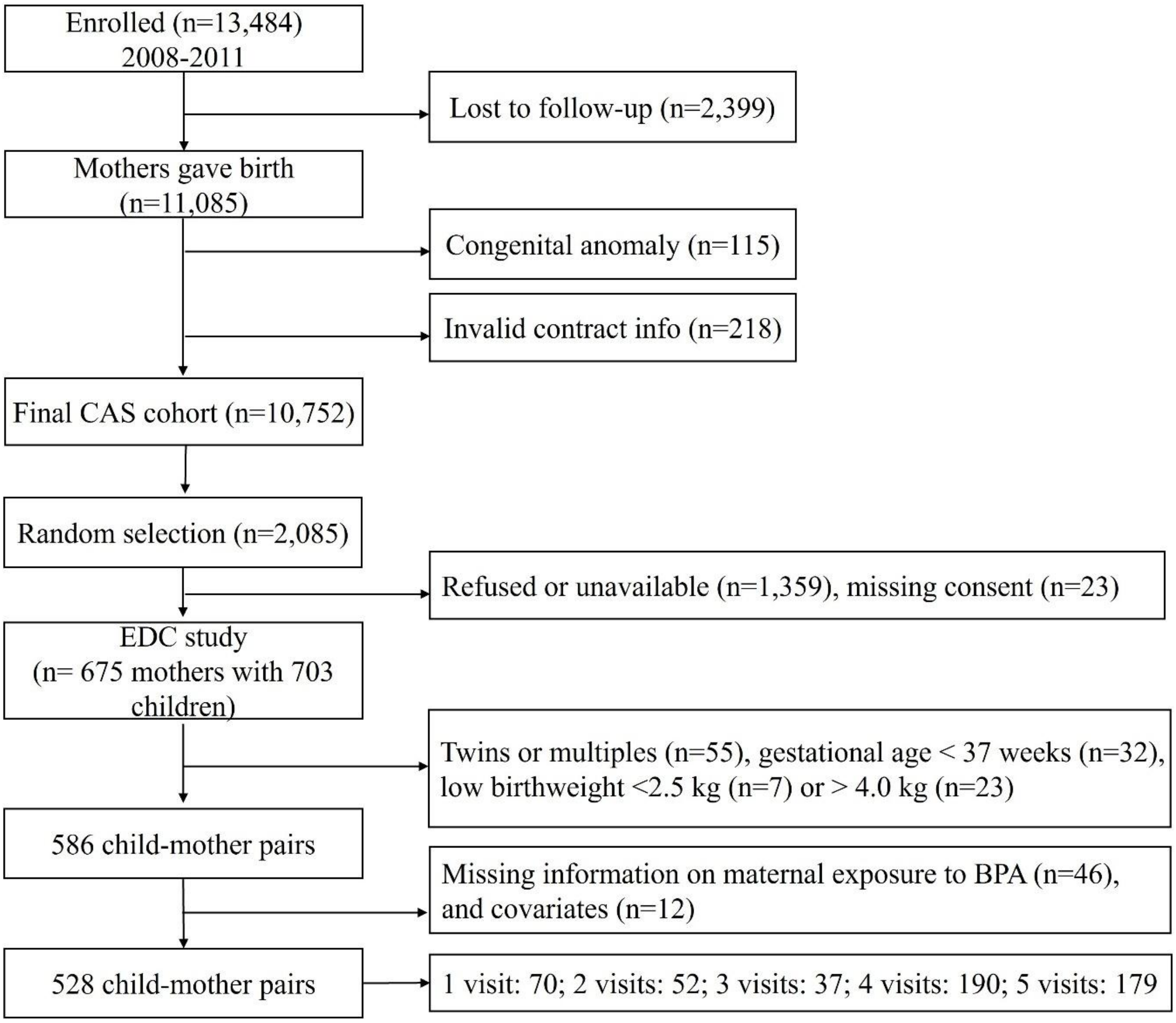

Number of children in each follow-up by birth year 2009 2010 2011

Age (years)	N	Follow-up year									
		2012	2013	2014	2015	2016	2017	2018	2019	2020	2021
2	338	227<br>5	1<br>105								
4	535		146	232<br>11	111						
6	481				153	3<br>213<br>10	2<br>100				
8	441						143	197	9<br>92		
10	373								127	164<br>24	4<br>54

CAS: Congenital Anomaly Study; EDC: Environment and Development of Children

Supplementary Material, Fig S2. Directed acyclic graph to determine covariates in the models (maternal exposure to bisphenol A [BPA] and body mass index [BMI])

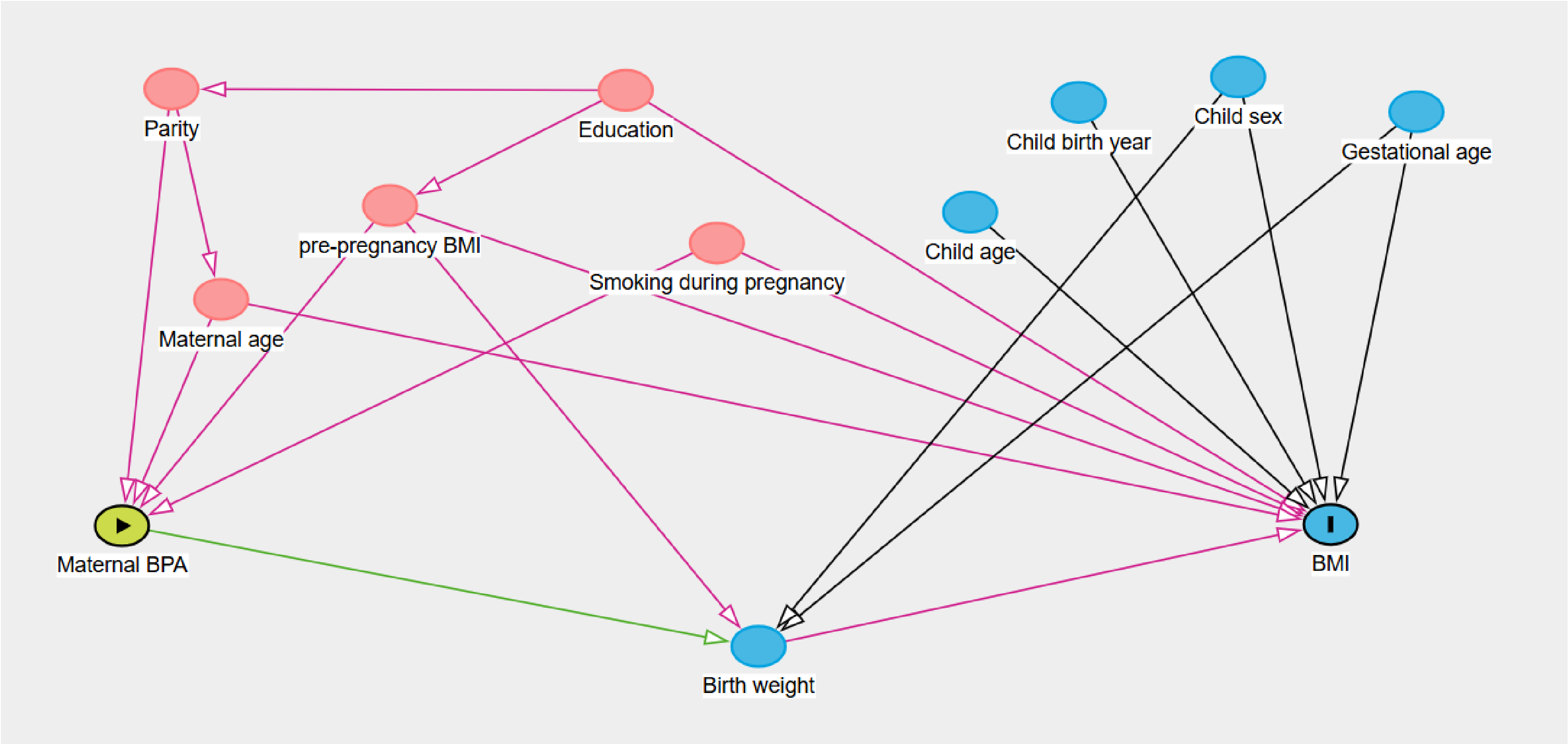

Supplementary Material, Fig S3. Sex-specific mean (a) BMI and (b) BMI-Z at each age by maternal BPA exposure groups (low [below median] and high [above median])

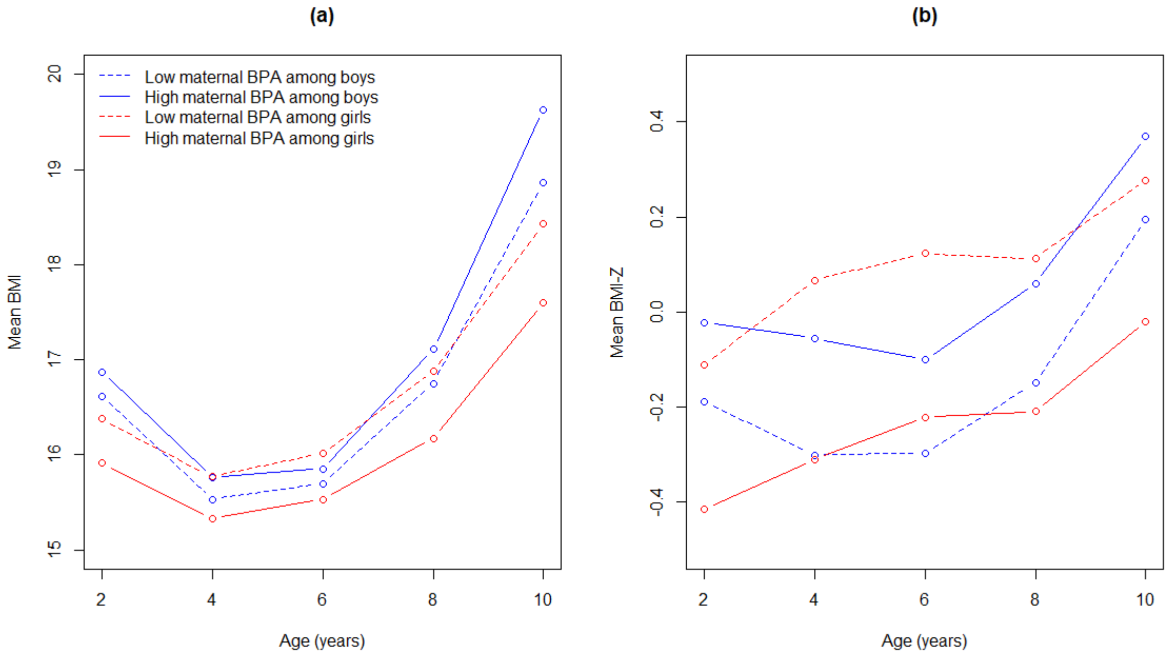

BMI, body mass index; BMI-Z, BMI Z-score; BPA, bisphenol A

Supplementary Material, Fig S4. Sex-specific differences in BMI and BMI Z-score at each age associated with quartile-based maternal BPA exposure (95% confidence intervals) among children (n = 528), with Q2–Q4 compared to Q1.

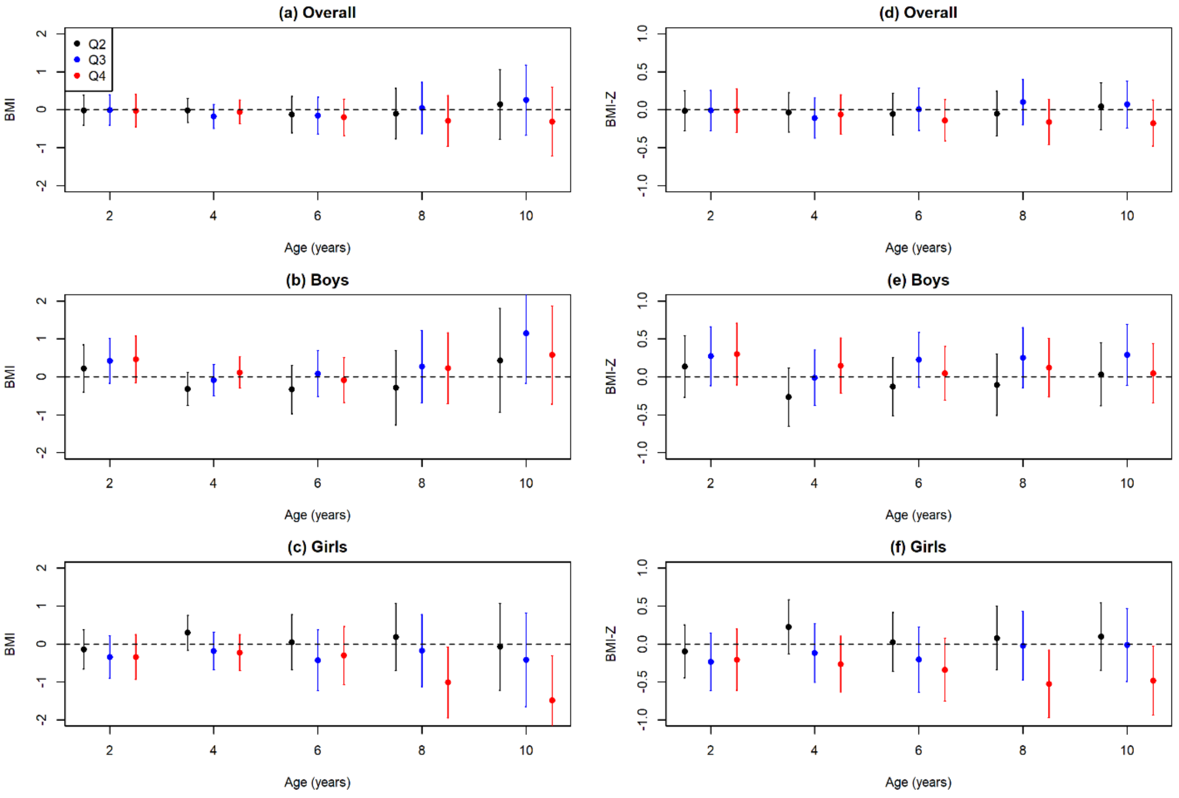

BMI, body mass index; BMI-Z, BMI Z-score; BPA, bisphenol A
Models were adjusted for maternal characteristics, including age, parity, pre-pregnancy BMI, educational attainment, and smoking status, as well as child characteristics such as birth year, age at visit (months), sex (available only in overall estimates), gestational age, and birth weight.

Supplementary Material, Table S1. Sex-stratified changes in BMI and BMI Z-score (95% Confidence Intervals) at each age associated with a one-unit increase in the log-transformed maternal BPA exposure (n=528), as shown in Figure 1

Outcome	Age (years)	Overall	Boys	Girls	P-value for sex difference[§]
BMI (kg/m^2)	2	0.04 (-0.12, 0.20)	0.21 (-0.02, 0.43)	-0.13 (-0.37, 0.10)	0.02
	4	-0.02 (-0.13, 0.09)	0.08 (-0.06, 0.22)	-0.12 (-0.29, 0.05)	0.04
	6	-0.04 (-0.21, 0.12)	0.02 (-0.18, 0.22)	-0.16 (-0.43, 0.11)	0.23
	8	-0.03 (-0.26, 0.21)	0.15 (-0.17, 0.47)	-0.30 (-0.63, 0.03)	0.04
	10	0.01 (-0.29, 0.32)	0.33 (-0.11, 0.77)	-0.42 (-0.82, -0.01)[*]	0.01
BMI Z-score	2	0.03 (-0.08, 0.14)	0.13 (-0.01, 0.28)	-0.08 (-0.24, 0.08)	0.03
	4	-0.01 (-0.10, 0.08)	0.09 (-0.04, 0.21)	-0.12 (-0.25, 0.02)	0.02
	6	-0.02 (-0.11, 0.08)	0.05 (-0.07, 0.17)	-0.13 (-0.27, 0.02)	0.04
	8	-0.01 (-0.11, 0.10)	0.08 (-0.05, 0.22)	-0.15 (-0.30, 0.01)	0.02
	10	-0.01 (-0.12, 0.09)	0.07 (-0.06, 0.21)	-0.13 (-0.29, 0.03)	0.03

The models were adjusted for maternal factors such as age, parity, pre-pregnancy BMI, educational attainment, and smoking status, along with child-related factors, including age, birth year, gestational age, and birth weight.

[*] P-value <0.05 for overall, boys, and girls; [§] P-values for sex differences in changes in BMI and BMI Z-score associated with maternal BPA exposure.

Supplementary Material, Table S2. Sex-stratified odds ratio of being overweight (95% Confidence Intervals) at each age associated with a one-unit increase in the log-transformed maternal BPA exposure (n=528)

Age	Overall	Boys	Girls	P-value for sex difference§
Overall	0.95 (0.84, 1.09)	1.07 (0.90, 1.28)	0.82 (0.68, 1.00)	0.05
2	0.99 (0.60, 1.65)	1.26 (0.66, 2.38)	0.78 (0.31, 1.96)	0.26
4	0.95 (0.72, 1.26)	1.16 (0.78, 1.72)	0.81 (0.54, 1.20)	0.16
6	0.95 (0.72, 1.27)	0.88 (0.58, 1.33)	1.03 (0.69, 1.54)	0.57
8	0.91 (0.69, 1.20)	0.98 (0.65, 1.47)	0.76 (0.50, 1.14)	0.48
10	0.96 (0.75, 1.24)	1.20 (0.86, 1.69)	0.70 (0.47, 1.06)	0.05

Overall and age-specific odds ratios were estimated in mixed and logistic regression models, respectively.
The models were adjusted for maternal factors such as age, parity, pre-pregnancy BMI, educational attainment, and smoking status, along with child-related factors, including age, gestational age, and birth weight. For postnatal BPA exposure, these adjustments were maintained with the addition of breastfeeding. Sex was included as a variable in the model for overall estimation.
§ P-values for sex differences in changes in BMI and BMI Z-score associated with maternal BPA exposure.

Supplementary Material, Table S3. Sex-stratified changes in longitudinal BMI and BMI Z-score (95% Confidence Intervals) associated with a one-unit increase in the log-transformed maternal BPA exposure, before and after adjusting for breastfeeding (n=528)

	Main analysis: Before adjusting for breastfeeding		Sensitivity analysis: After adjusting for breastfeeding	
Sex	BMI (kg/m^2)	BMI Z-score	BMI (kg/m^2)	BMI Z-score
Overall[a]	-0.01 (-0.10, 0.08)	-0.01 (-0.05, 0.04)	0.00 (-0.09, 0.09)	-0.01 (-0.05, 0.04)
Boys[a]	0.15 (0.03, 0.27)*	0.08 (0.02, 0.14)*	0.16 (0.03, 0.28)*	0.08 (0.02, 0.14)*
Girls[a]	-0.20 (-0.33, -0.07)*	-0.12 (-0.18, -0.05)*	-0.20 (-0.33, -0.07)*	-0.11 (-0.18, -0.05)*

BMI, body mass index; BMI-Z, BMI Z-score; BPA, bisphenol A

[a] Associations for overall ages were analyzed using linear mixed models.

[*] P-value <0.05

Models in the main analysis were adjusted for maternal characteristics, including age, parity, pre-pregnancy BMI, educational attainment, and smoking status, as well as child characteristics such as birth year, age at visit (months), sex (available only in overall estimates), gestational age, and birth weight. Sensitivity analyses further adjusted for breastfeeding in addition to the covariates included in the main models.

Supplementary Material, Table S4. Sex-stratified mediation proportion by birth weight on the association between maternal BPA exposure and BMI at each age

Sex	Age (years)	Mediation proportion (%, 95% Confidence Limits)	P-value
Boys	2	-2.1 (-39.1, 16.1)	0.78
	4	24.1 (-180.1, 243.1)	0.24
	6	36.1 (-207.1, 159.1)	0.81
	8	11.1 (-123.1, 108.1)	0.63
	10	2.1 (-28.1, 32.1)	0.91
Girls	2	25.1 (-92.1, 226.1)	0.27
	4	0.1 (-190.1, 160.1)	0.90
	6	2.1 (-137.1, 163.1)	0.83
	8	1.1 (-28.1, 31.1)	0.83
	10	3.1 (-19.1, 28.1)	0.63
